# Optical Properties, Morphology, and Stability of Iodide-Passivated Lead Sulfide Quantum Dots

**DOI:** 10.3390/ma12193219

**Published:** 2019-10-01

**Authors:** Ivan D. Skurlov, Iurii G. Korzhenevskii, Anastasiia S. Mudrak, Aliaksei Dubavik, Sergei A. Cherevkov, Petr S. Parfenov, Xiaoyu Zhang, Anatoly V. Fedorov, Aleksandr P. Litvin, Alexander V. Baranov

**Affiliations:** 1 Center “Information Optical Technologies”, ITMO University, 49 Kronverksky Pr., St. Petersburg 197101, Russiaa_v_fedorov@mail.ifmo.ru (A.V.F.); litvin@itmo.ru (A.P.L.); a_v_baranov@mail.ifmo.ru (A.V.B.); 2 College of Materials Science, Jilin University, Changchun 130012, China

**Keywords:** quantum dots, lead sulfide, ligand exchange, iodide, stability

## Abstract

Iodide atomic surface passivation of lead chalcogenides has spawned a race in efficiency of quantum dot (QD)-based optoelectronic devices. Further development of QD applications requires a deeper understanding of the passivation mechanisms. In the first part of the current study, we compare optics and electrophysical properties of lead sulfide (PbS) QDs with iodine ligands, obtained from different iodine sources. Methylammonium iodide (MAI), lead iodide (PbI_2_), and tetrabutylammonium iodide (TBAI) were used as iodine precursors. Using ultraviolet photoelectron spectroscopy, we show that different iodide sources change the QD HOMO/LUMO levels, allowing their fine tuning. AFM measurements suggest that colloidally-passivated QDs result in formation of more uniform thin films in one-step deposition. The second part of this paper is devoted to the PbS QDs with colloidally-exchanged shells (i.e., made from MAI and PbI_2_). We especially focus on QD optical properties and their stability during storage in ambient conditions. Colloidal lead iodide treatment is found to reduce the QD film resistivity and improve photoluminescence quantum yield (PLQY). At the same time stability of such QDs is reduced. MAI-treated QDs are found to be more stable in the ambient conditions but tend to agglomerate, which leads to undesirable changes in their optics.

## 1. Introduction

Type of the ligand and exchange treatment procedure govern quantum dot (QD) solid properties, such as energy structure, charge transport, and stability [1,2]. Long organic ligands, commonly used in organometallic QD synthesis, lead to large interdot distances after the film deposition. Due to such distances, dot-to-dot coupling is reduced and charge transfer between QDs vanishes [3]. Therefore, QD film acts as an insulator, preventing its application as an active layer in optoelectronic devices. One of the ways to increase QD coupling is to reduce interdot spacing by ligand stripping or ligand exchange (LE). During LE, it is also possible to reduce the number of surface defects responsible for non-radiative carrier losses.

These days, LE can be performed with different methods and compounds [4]. The most common substitute for an initial oleate shell around PbS QDs is short organic ligands that have functional groups which can strongly bind to Pb atoms on the QD surface (e.g., thiols, amines) [5,6]. Another promising approach is the use of QD perovskite shelling, due to perovskites’ small lattice mismatch with PbS QDs [7,8,9]. Molecular metal chalcogenide ligands, introduced by Talapin’s group [10,11], have allowed fabricating efficient field effect transistors and photodetectors with considerably enhanced carrier mobility and conductivity. Atomic LE is similar to the previous method and allows the formation of an atomic shell with a high binding energy to the QD surface [12]. Halide passivation has allowed the fabrication of efficient and stable QD-based devices [13]. Within the halides, iodide atoms tend to form the strongest bond with the PbS QD surface [14]. Such passivation may also occur during PbSe QD synthesis via cation exchange, when residual halide atoms act as atomic passivating layer [14].

One strategy is post-deposition (or so-called solid-state) LE, which strips the existing oleate ligands, simultaneously binding the desired new ligand (e.g., tetrabutylammonium iodide (TBAI), ethanedithiol) to the exposed QD surface [4,15]. However, during solid-state LE, cracks may from on the surface of the QD film. The cracks are formed because of the native ligand volume loss during their stripping, leading to the low charge transfer rate [3]. To compensate this, several steps of layer-by-layer deposition are required. The whole procedure results in the increase of wasted material. To avoid it, colloidal phase LE was introduced. After the deposition step, such QD films have reduced interdot distances and uniform surface. With these improvements, higher coupling and charge transfer rate can be achieved. The two aforementioned methods can be combined as a hybrid passivation, i.e., solution phase halide LE followed by a solid-state treatment with short-chain organic molecules [16]. Utilizing PbS QD with both direct and hybrid colloidal iodide treatment, efficiencies of up to 12% have been achieved [17,18,19,20].

When QDs are used in optoelectronic devices, their environmental stability is of exceptional importance. Due to the high surface-to-volume ratio of the QDs, they become very susceptible to environment impact, which leads to undesired changes of QD properties. The lack of the degradation theory makes these changes unpredictable. Because of the lead-rich surface [21,22,23], oxide molecules can be formed on a QD surface even with ~1 ppm of O_2_ molecules [3]. A small amount of oxygen induces p-type doping of PbS QDs [24] and creates additional recombination centers. A thicker oxide layer changes the dielectric confinement and reduces the effective diameter of the QD core, which increases their bandgap. Such a change can alter the device architecture, leading to unbalanced charge separation and transfer. As a result, the devices lose their efficiency. A thorough ligand-engineering promotes better stability of QD solids and devices.

Despite the high demand of halide LE, there is a lack of direct comparison between different iodide treatments. In this paper, we investigate the impact of iodide source on the PbS QDs’ optical and electronic properties. We show that the use of methylammonium iodide (MAI), lead iodide (PbI_2_), and TBAI have both advantages and disadvantages, indicating that a careful choice of both the LE procedure and the iodide source is required for different QD applications. Furthermore, we investigate how colloidal ligand exchange affects QD aging, both in colloidal and solid-state form.

## 2. Materials and Methods

### 2.1. QD Synthesis.

Lead sulfide QDs with an average diameter of 4 nm were synthesized according to Ushakova et al. [25]. In short, sulfur precursor (hexamethyldisilthiane) in octadecene was injected into the three-neck flask, containing pre-heated (112 °C) solution of PbO in octadecene and oleic acid (OA). After one min, the reaction mixture was cooled down, then QDs were precipitated with methanol and redispersed in n-hexane for further processing.

### 2.2. Ligand Exchange.

Colloidal ligand exchange with lead iodide (PbI_2_) was performed as reported in [26]. Briefly, PbS QD solution in toluene was added to the PbI_2_ solution in the mixture of dimethylformamide (DMF) and n-butylamine (BA). To achieve LE, the resulting solution was shaken for two min. Precipitated QDs were redispersed in 1,2-dichlorobenzene (DCB) and BA mixture for deposition and storage. Colloidal MAI treatment was adopted from Lan et al. [18]. First, 1.5 mL of MAI solution in the mixture of toluene and DMF were added drop-by-drop to the 2 mL PbS QD solution in toluene and then softly shaken. The resulting homogeneous solution was left for 12 h to complete the exchange. For film deposition, QDs were precipitated with methanol and redispersed in octane. Post-deposition LE with TBAI was carried out according to Lu et al. [27]. TBAI solution in methanol was applied to the spin-coated OA-capped PbS QD film. Then, the excess of TBAI, as well as remaining native oleic acid ligands, were removed with subsequent washing with acetonitrile [27,28].

### 2.3. Samples Preparation.

OA-, PbI_2_-, and MAI-capped PbS QD thin films were spin-coated from ∼30 mg/mL colloidal solutions on a glass substrate at 2500 RPM for 1 min. Substrates were washed with acetone and plasma-cleaned prior to the deposition. For UPS measurements, ∼20-nm-thick QD film was spin-coated onto ITO-covered glass substrate. For the conductivity and frequency capacitance measurements, QDs were spin-coated onto the patterned ITO substrates.

QD stability in the ambient atmosphere was studied for both QD solids and QD solutions. The reference sample of OA-capped QDs was dissolved in tetrachloromethane; the MAI-treated QDs were dissolved in a mixture of toluene and DMF (4:1 volume); and PbI_2_-treated QDs were dissolved in a mixture of DCB and BA (5:1 volume). All solutions were prepared with concentration of ∼10^−6^ M. Sartorius filter paper (388 grade, pore diameter >20 µm) was used as a porous matrix. QDs were transferred to the porous matrix via drop-casting the diluted solution (concentration of 10^−6^ M) onto the matrix. From this moment on, QDs in porous matrix will be referred to as ‘QD solids’. All of the samples were stored in ambient conditions without any light exposure.

### 2.4. Characterization

Fourier-transformed infrared (FTIR) spectra were acquired using Tensor 27 FTIR spectrometer (Bruker, Germany). Atomic force microscopy (AFM) measurements were taken with Solver-Pro (NT-MDT, Russia) atomic force microscope in the semicontact mode. UV-VIS-NIR absorption spectra were acquired using Shimadzu UV3600 spectrophotometer (Shimadzu, Japan) equipped with the Shimadzu ISR-3100 integration sphere. The ultraviolet photoelectron spectra (UPS) were collected using PREVAC UPS system (PREVAC, Poland). QD HOMO level was determined directly from the UPS measurements.LUMO energy was calculated by adding QD bandgap energy to HOMO value. QD bandgap was determined from the position of the first excitonic absorption peak [25], adding the Coulomb stabilization energy (53 meV in case of 4 nm PbS QDs) [1]. Conductivity was acquired from I-V curves taken with Tektronix Keithley 2636 source meter (Tektronix, USA). QD states energy distribution (density of states) was found from the frequency capacitance (C-f) characteristics, obtained with Keysight E4980A Precision LCR Meter (Keysight, USA). Transient and time-resolved photoluminescence measurements were taken with custom-built setup for NIR-PL detection; the detailed information about the setup is available elsewhere [29]. Quantum yield was measured against the standard IR dye with the known photoluminescence quantum yield (PLQY). Sigma-Aldrich IR1061 dye (Merck, Germany) with a PLQY of 1.8% was dissolved in dichloromethane and used as a reference sample. To evaluate the stability for both solutions and solids, regular measurements of PL spectra and PL decay kinetics were performed during 35 days of storage. Assuming that PL peaks had gaussian form, the measured spectra were approximated and the values for peak position and FWHM were extracted.

## 3. Results and Discussion

### 3.1. Iodine Source Impact on the PbS QD Parameters

**Figure 1:**
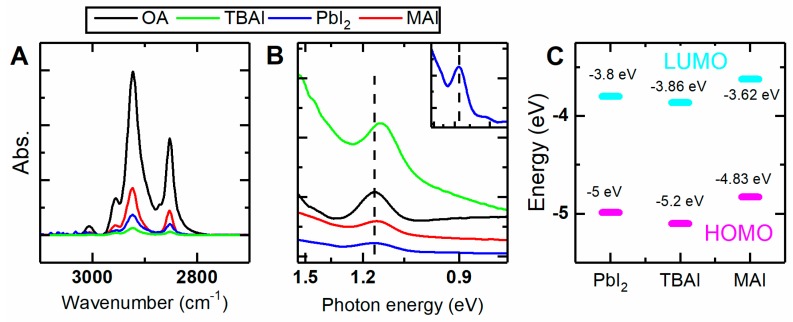
(**A**) FTIR absorption spectra of quantum dot (QD) colloidal solution. Black, OA-capped QDs; green, TBAI-treated QDs; blue, PbI_2_-treated QDs; red, MAI-treated QDs. (**B**) QD thin film absorption spectra (offset for clarity); on inset, PbI_2_-treated QD thin film absorption. (**C**) HOMO (purple dashes) and LUMO (cyan dashes) energy levels of iodide-treated PbS QD thin films.

LE efficiency was estimated by monitoring the residual OA ligands on the QD surface using the FTIR spectroscopy. OA had distinguishable absorption bands in the range of 3000–2800 cm^−1^_,_ corresponding to the C–H stretching oscillations. FTIR absorption spectra for OA-, TBAI-, MAI-, and PbI_2_-passivated QD thin films are presented in Figure 1A. To take into the account the number of the QDs in the studied films, the obtained FTIR spectra were normalized to a corresponding film absorption at their fundamental absorption band (600 nm). FTIR spectra show that most of the OA molecules were displaced from the QD surface. MAI treatment displaced about 80% of the native OA ligands. Exchange efficiencies for PbI_2_ (87%) and TBAI (95%) treatments correspond well to the published protocols [26,27].

Absorption spectra obtained for the QD films are presented in Figure 1B. Films made from QDs with colloidal LE (MAI, PbI_2_) demonstrated negligible changes in their absorption spectra (blue and red lines on Figure 1B). TBAI-treatment forms densely-packed film (see below), intensifying the dipole interactions between QDs, which led to a red-shift (40 meV) in the absorption band, as it was recently shown [30,31].

QDs with different ligand shells have different positions of the conduction band bottom (LUMO) and the valence band top (HOMO) [1]. We show that the choice of the iodine source also affects the HOMO/LUMO levels. Application of different iodine sources changed the HOMO/LUMO energies for up to 0.37 eV (see Figure 1C). We believe that such changes are supposed to be interpreted in terms of QD stoichiometry. PbS QDs are mostly non-stoichiometric due to their high surface-to-volume ratio and the exposure of lead-rich (111) facet [21,22]. Density functional theory calculations show that passivation of the uncompensated surface Pb atoms leads to the elimination of the mid-gap states and shifts the HOMO/LUMO energies [32]. Choice of iodine source changes the amount of iodine attached to the QD surface, as has been determined by means of XPS and NMR spectroscopy [17,18]. This fact leads to different amounts of uncompensated Pb atoms on the surface of the QD, which alters the QD stoichiometry. Consequently, different procedures of iodide passivation allow fine control of the QD’s energy levels, which is of drastic importance for optoelectronic applications.

**Figure 2:**
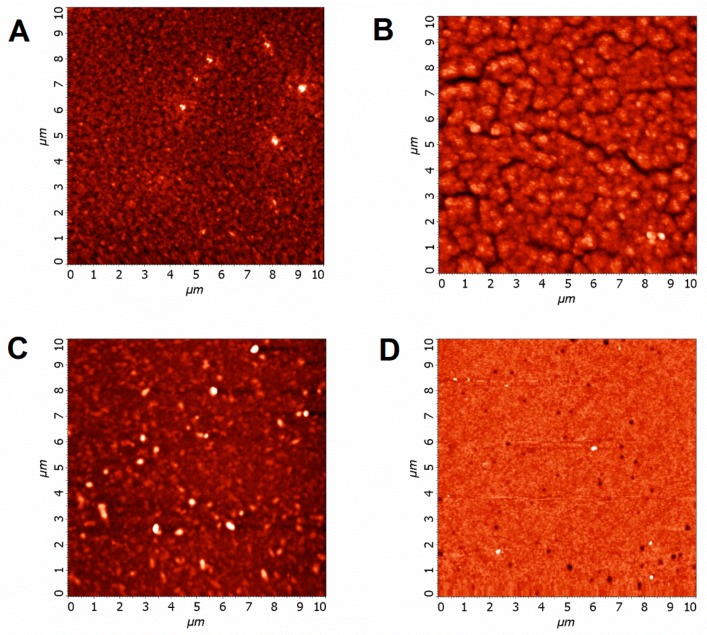
AFM images (10 × 10 µm) of spin-cast PbS QDs (d = 4 nm) films: (**A**) With native oleic acid capping; (**B**) after TBAI solid-state treatment; (**C**) after colloidal MAI-treatment; (**D**) after colloidal PbI_2_ treatment.

Morphology and thickness of films were determined through AFM measurements (Figure 2). To estimate the surface uniformity quantitatively, we employed an averaged surface roughness as a figure of merit (values are listed in Table 1). In high-quality film, averaged roughness should not exceed QD diameter (about 4 nm in our case). The film made from OA-capped QDs (see Figure 2A) had highly varying roughness values that were scattered in the range of 3–17 nm, with an averaged value of 10 nm. Such poor reproducibility of the experimental results can be partly attributed to the interaction between long OA chains and AFM probe. Generally, all iodide treatments presented resulted in smoother film surface, compared with the film made from OA-capped QDs. Solid-state TBAI treatment led to the formation of the cracks on the QD film surface (Figure 2B). These cracks were formed due to the ligand volume loss during the post-deposition LE and can be mitigated with layer-by-layer deposition. Despite the presence of the cracks, TBAI-treated films are still more uniform than the OA-capped ones, judging from the reproducible roughness values of ~7 nm. Films made from MAI-treated QDs (Figure 2C) contain QD aggregates but, due to the absence of the cracks, they have an averaged roughness of 6 nm, which is close to the TBAI-treated sample. In their turn, films made from PbS QDs with PbI_2_ LE demonstrate almost no defects, as can be seen in Figure 2D. An averaged roughness of such films does not exceed 2 nm, which indicates highly uniform film. We compared the packing density of the films by dividing the optical density at the fundamental absorption band (at 600 nm) to the corresponding film thickness. Such figure of merit characterizes the packing density. Ligand-exchanged treatments in ascending order of resulting packing density: Native OA capping, MAI, PbI_2_, TBAI (see Table 1 for the figure of merit values).

Electrophysical properties of ligand-exchanged QD films were compared by analysis of film resistivity and density of trap-states (DoS) (see Table 1 for details). OA-capped QD films behave as an insulator. The MAI-treated sample has the worst resistivity of ~2.5 MOhm·m, while films made from TBAI- and PbI_2_-treated QDs have resistivity values close to each other: 7 kOhm·m and 12 kOhm·m, respectively. Such values can be explained through the difference in the films’ packing density. The more densely-packed TBAI-treated sample has the lowest resistivity, while the resistivity of the thinner-packed MAI-treated sample is higher by an order of magnitude. Originally, MAI treatment was developed to be combined with solid-state TBAI treatment [18]. In the absence of the latter, some residual organic MA^+^–oleate complexes might reside inside of the film, increasing interparticle distance. Consequently, charge transfer within film is hindered. This, coupled with the observed aggregation, leads to the rather small change in the MAI-film resistivity under AM1.5 irradiation: From 2.5 MOhm·m to 1 MOhm·m. In turn, both TBAI- and PbI_2_-treated films demonstrate high photoconductive responses, indicating the efficient charge transfer in these films.

It has been shown [33,34] that so-called deep trap states (with energy >0.2 eV below the conduction zone) within the QD film greatly influence the resulting device performance. Our DoS data (Figure S1) reveal that MAI- and TBAI-treated QDs have similar amounts of such deep traps (~4·10^18^ eV^−1^·cm^−3^). As we have already shown, MAI-treated QDs tend to agglomerate, which increases the carrier chance to be trapped in the nonradiative state. PbI_2_ treatment, in turn, reduces the number of deep traps by an order in comparison with TBAI and MAI treatments. We interpret this as a sign of a more efficient QD surface passivation.

### 3.2. Optical Properties of QD Solutions After the Colloidal LE

**Figure 3:**
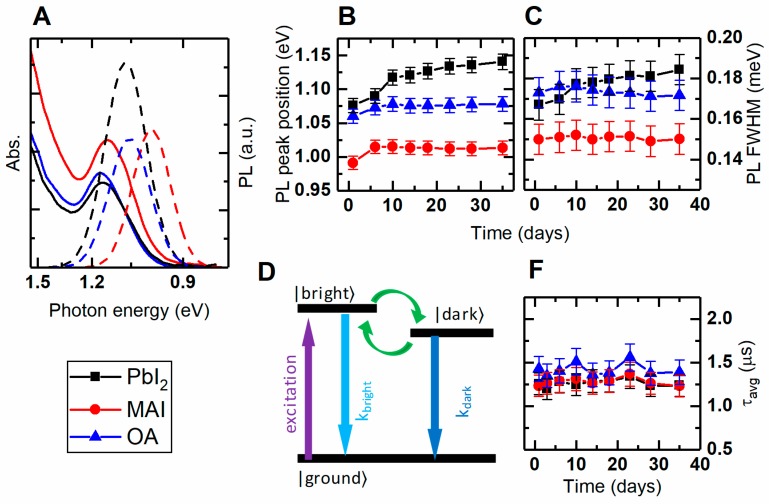
(**A**) Optical absorption and PL of colloidal PbS QDs with different ligand shell. Black, PbI_2_; red, MAI; blue—OA. (**B**) PL peak position evolution; (**C**) PL full width at half maximum evolution; (**D**) energy level model diagram; (**E**) PL averaged decay time evolution.

Absorption and NIR-PL spectra of as-prepared and ligand-exchanged colloidal PbS QDs are shown in Figure 3A. All of the studied QDs absorb throughout UV-NIR spectral region up to 0.88 eV (see Figure 1B and Figure 2A). Absorption and emission parameters for colloidal samples are summarized in Table 2. In comparison with the OA-capped QDs, PbI_2_-treated QDs have slightly blue-shifted emission and absorption maxima, reduced Stokes shift, and increased PLQY (see Table 2 for details). The two latter facts indicate the reduction in the amount of non-radiative recombination centers due to more effective passivation of surface trap states. This also correlates with the reduction of the deep traps from the DoS measurement. MAI-treated QDs are red-shifted in both absorption and emission, with reduced PL FWHM. However, due to the increased Stokes shift and reduced PLQY, we believe that the main reasons of such behavior are the self-absorption and nonradiative recombinations in QD agglomerates (see AFM in the section above). The former arises from the large amount of sub-bandgap states which are more pronounced in MAI-treated QDs, as evident from the more pronounced absorption low-energy tail (see Figure 3A, red solid line).

### 3.3. QD Aging after the Colloidal LE

Energy level structure and its evolution can be accurately monitored with PL spectroscopy, since it is more sensitive than the absorption methods. However, when QD solids are a subject of PL study, Förster resonant energy transfer (FRET) should be taken into account. Due to a certain size distribution, FRET can occur even in a quasi-monodisperse QD ensemble [35,36]. It leads to a red shift of PL peak position and PL dynamics modification. To carefully analyze the PL peak shape and position along with decay rates, FRET must be eliminated. It has been recently shown that FRET can be neglected if QDs are inserted into a porous matrix at comparatively low QD concentration [37]. Hence, QDs were embedded into a porous matrix at the concentration of 6 × 10^15^ cm^−3^. Changes in the PL parameters for both colloidal and solid-state samples are summarized in Table S1.

#### 3.3.1. QD Colloidal Solutions

**Figure 4:**
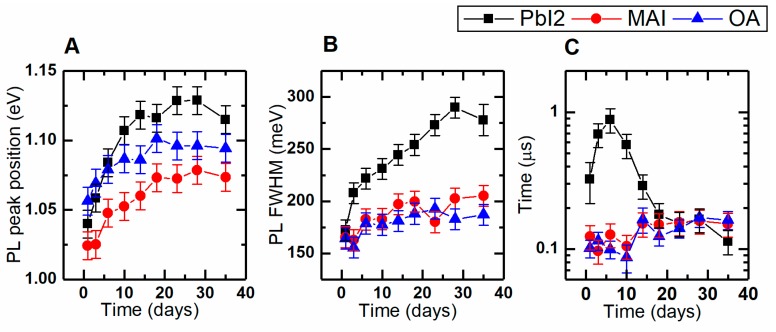
Colloidally-exchanged QDs aging in porous matrix (black squares, PbI_2_-treated QDs; red circles, MAI-treated QDs; blue triangles, OA-capped QDs). (**A**) PL peak positions; (**B**) PL full width at half maximum; (**C**) PL averaged decay time.

Evolution of the PL parameters of the colloidal QDs is displayed in Figure 3. All colloidal QDs demonstrate a noticeable change in PL peak position during the first 6–10 days after synthesis and exchange (Figure 3B). We relate this change to the interactions with the oxygen and moisture from the ambient air. Apart from the first days, the OA-capped and MAI-treated samples (blue triangles and red circles in Figure 4, respectively) demonstrate a high stability of their PL responses. PbI_2_-treated QDs are less stable and blue-shifting over the time of storage (Figure 3B, black squares). Blue shift is the sign of QD etching and reduction in QD size. One of the reasons for such a behavior might be the presence of n-butylamine as a cosolvent. Due to the incomplete iodide capping of the QD, there are places where BA could attach to the QD surface. It has been previously noticed that BA negatively affects the colloidal stability and PL properties of lead sulfide QDs [26,38]. To prove it, we redispersed PbI_2_-treated QDs in pure n-butylamine and monitored their PL evolution (see Figure S2). During the first 6 days, QD PL maximum drastically changed from 1.075 to 1.23 eV, accompanied with FWHM increase from 170 to 220 meV. On the 10th day, QDs were completely dissolved by BA. In contrast to the spectral parameters, averaged PL decay times obtained for colloidal solutions show no notable evolution (see Figure 3E). A negligible change in decay kinetics over the time of storage can be attributed to the small change of the QD effective size.

#### 3.3.2. QDs in Porous Matrix

The QD degradation is more pronounced in the solid-state form due to the direct contact with atmospheric oxygen (see Figure 4A). QDs located closer to the surface are more prone to interaction with the environment. Their properties change faster, leading to a significant broadening of PL spectra [37], represented as PL FWHM increase in Figure 4B. Both the OA-capped and the MAI-treated QDs demonstrate similar behavior in PL spectral, parameters with QD–MAI having slightly less pronounced PL peak shift. As in colloidal form, PbI_2_-treated QDs are also less stable in porous matrix. Such samples demonstrate a dramatic shift of PL peak position (Figure 4A—black squares) and a dramatic increase in FWHM (Figure 4B—black squares). This could be explained through the presence of the residual BA bound to the QD surface in the solution phase, which had already been proven to be detrimental for the QDs.

In contrast to their colloidal counterparts, QD solids suffer from a noticeable change of their PL decay during storage (Figure 4C). Averaged decay time is not enough to understand the underlying mechanisms of QD aging. There has been multiple evidences of excitonic fine structure in PbS QDs [25,39,40]. Based on these evidences, we used a three-level model of the PbS QD energy structure (see Figure 3D) with two radiative states and the ground state. One state with the high radiative decay rate was labeled as ‘bright’ and another one with the lower radiative decay rate was labeled as ‘dark’ (see Supplementary materials for the detailed information about the decay parameters calculation). Evolution of the bright/dark decay times and relative amplitudes is presented in Figure S3. For the OA-capped sample, we observe a stable increase in the averaged decay time (Figure 4C—blue triangles). We believe that there are two processes taking place: The first—increase in the relative amount of ‘dark’ excitonic states with longer radiative lifetime (Figure S3A) due to increase in the amount of traps during the QD prolonged air exposure. The second – slight increase in both ‘dark’ and ‘bright’ state lifetimes (Figure S3D). The latter can be explained through the reduction in effective QD size, followed by the increase in PL lifetime [25]. The MAI-treated sample demonstrates similar changes in PL decay times (Figure S3E), with an almost constant ratio of the ‘bright’/’dark’ states contribution (Figure S3B), indicating the better surface passivation. The PbI_2_-treated sample demonstrates unusual behavior in PL decay evolution (see Figure 4C—black squares; Figure S3C,F). Within 6 days after LE, we observed an increase in the PL lifetime, which was followed by its fast decrease. The reason of this peculiar behavior is yet to be determined, since there can be multiple factors influencing PbI_2_-treated QDs. These factors are the residual presence of n-butylamine, atmospheric oxygen, and the amount of iodide molecules on the QD surface.

## 4. Conclusion

To summarize, we performed a comparative study of iodide-passivated PbS QDs, prepared as both colloidal solutions and solids. We analyzed the influence of LE on the optical, electronic, and morphological properties of QD solids, as well as on the stability of QD properties. We show that more convenient and material-efficient colloidal MAI and PbI_2_ treatments result in QD film morphology and conductivity comparable to those achieved with common TBAI solid-state exchange. We found that PbI_2_-treated QDs demonstrate the highest PLQY and conductivity due to lower amount of the deep-trap states of as-exchanged QDs. As a drawback, PbI_2_-treated QDs possess poor environmental stability due to interactions with BA. In contrast, MAI-treated QDs show the highest stability of their PL responses, which is beneficial for creating more stable optoelectronic devices. These results indicate that a careful choice of LE procedure is required for each application. To achieve both high performance and stability, it is necessary to further develop LE techniques.

## Figures and Tables

**Table 1:** Parameters of QD films.

Shell Type	LE eff-cy	E_exc_, eV	Thickness, nm	Roughness, nm	Packing Density, a.u.	Dark Resistivity, kOhm*m	AM1.5 Resistivity, kOhm*m
Oleic acid	-	1.155 ± 0.005	110 ± 11	10 ± 7	0.96	-	-
PbI_2_	87%	1.165 ± 0.005	30 ± 3	2 ± 1	1.33	12 ± 2	0.31 ± 0.04
MAI	80%	1.142 ± 0.005	60 ± 6	7 ± 2	1.08	2500 ± 50	1000 ± 50
TBAI	95%	1.13 ± 0.005	160 ± 16	6 ± 2	2.75	7 ± 1	0.19 ± 0.02

**Table 2:** Optical parameters of colloidal PbS QDs.

Shell Type	E_exc_, meV	E_PL__,_ eV	FWHM, meV	Stokes Shift, meV	PLQY
PbI_2_	1155 ± 5	1075 ± 5	170 ± 7	80	~0.4
MAI	1125 ± 5	990 ± 5	130 ± 7	130	~0.13
Oleic acid	1170 ± 5	1060 ± 5	165 ± 7	110	~0.2

## Supplementary Materials

The following are available online at https://www.mdpi.com/1996-1944/12/19/3219/s1, Figure S1: Densities of trap states for QD with different LE, red line and circles—MAI-treated QDs, green line and triangles—TBAI-treated QDs, black line and squares—PbI2-treated QDs, Figure S2: Peak position shift of the PbI_2_-treated PbS QD, dispersed in pure n-butylamine, Figure S3: Colloidally exchanged QDs PL decay components evolution in porous matrix (black squares—PbI_2_-treated QDs, red circles—MAI-treated QDs, blue triangles—OA-capped QDs). Open symbol stands for relaxation from the bright state while solid symbol stands for relaxation from the dark state. Black lines are given as a guide to the eye; Table S1: Shifts of spectral PL parameters during 35-day storage in ambient conditions.
