# Supplementary material for: Optical Properties, Morphology, and Stability of Iodide-Passivated Lead Sulfide Quantum Dots
Source: Materials (Basel). 2019 Oct 1;12(19):3219. doi: 10.3390/ma12193219 (PMC6803903; doi:10.3390/ma12193219)
Supplement: Supplementary file 1 [file materials-12-03219-s001.pdf]

materials

Supplementary files

Optical Properties, Morphology and Stability of Iodide-Passivated Lead Sulfide Quantum Dots

Ivan D. Skurlov [1,*], Iurii G. Korzhenevskii [1], Anastasiia S. Mudrak [1], Aliaksei Dubavik [1], Sergei A. Cherevkov [1], Petr S. Parfenov [1], Zhang Xiaoyu [2], Anatoly V. Fedorov [1], Aleksandr P. Litvin [1] and Alexander V. Baranov [1]

[1] Center "Information Optical Technologies", ITMO University, 49 Kronverksky Pr., St. Petersburg 197101, Russia
[2] College of Materials Science, Jilin University, Changchun 130012, China
* Correspondence: ivan.skurlov.23@gmail.com

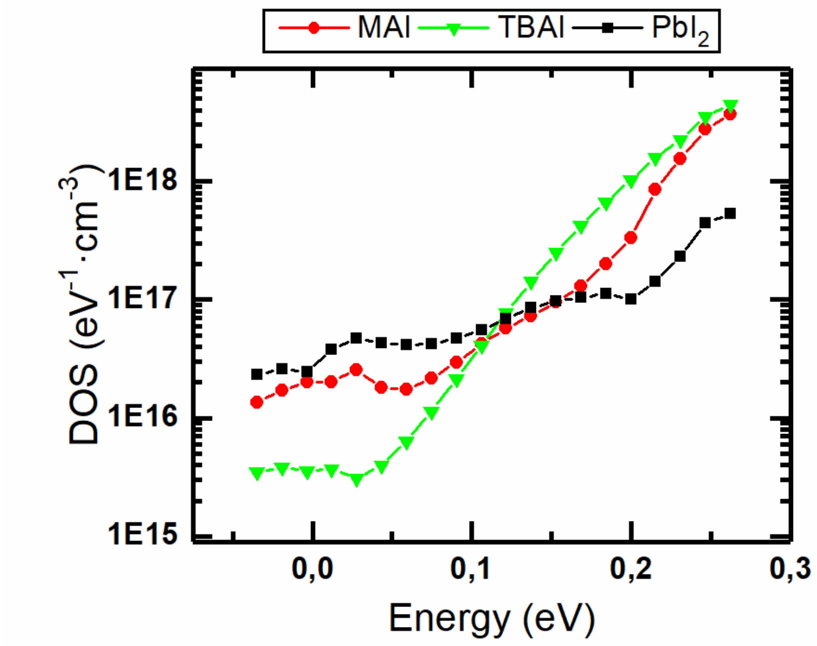

Figure S1. Densities of trap states for QD with different LE, red line and circles—MAI-treated QDs, green line and triangles—TBAI-treated QDs, black line and squares—PbI_2-treated QDs.

According to the model used, PL decay curves are approximated with a biexponential decay function:

$$I = A_D \exp(t/\tau_D) + A_B \exp(t/\tau_B),$$

$$\tau_{avg} = \frac{\sum A_i \tau_i}{\sum A_i}$$

where A_i—is a contribution from the state to the PL; τ_i—is radiative lifetime of the state; indices D and B define these components as 'dark' and 'bright' states, respectively.

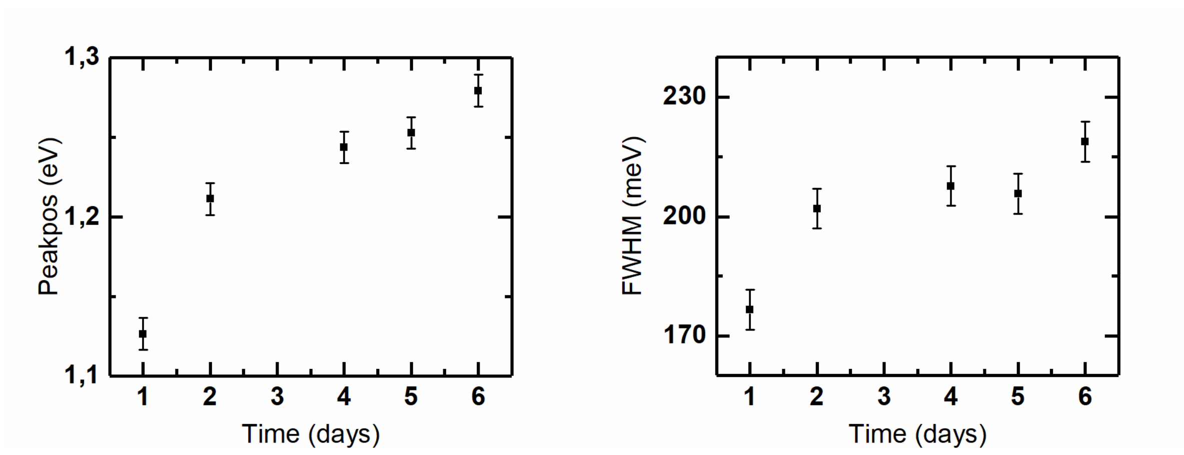

Figure S2. Peak position shift of the PbI_2-treated PbS QD, dispersed in pure n-butylamine.

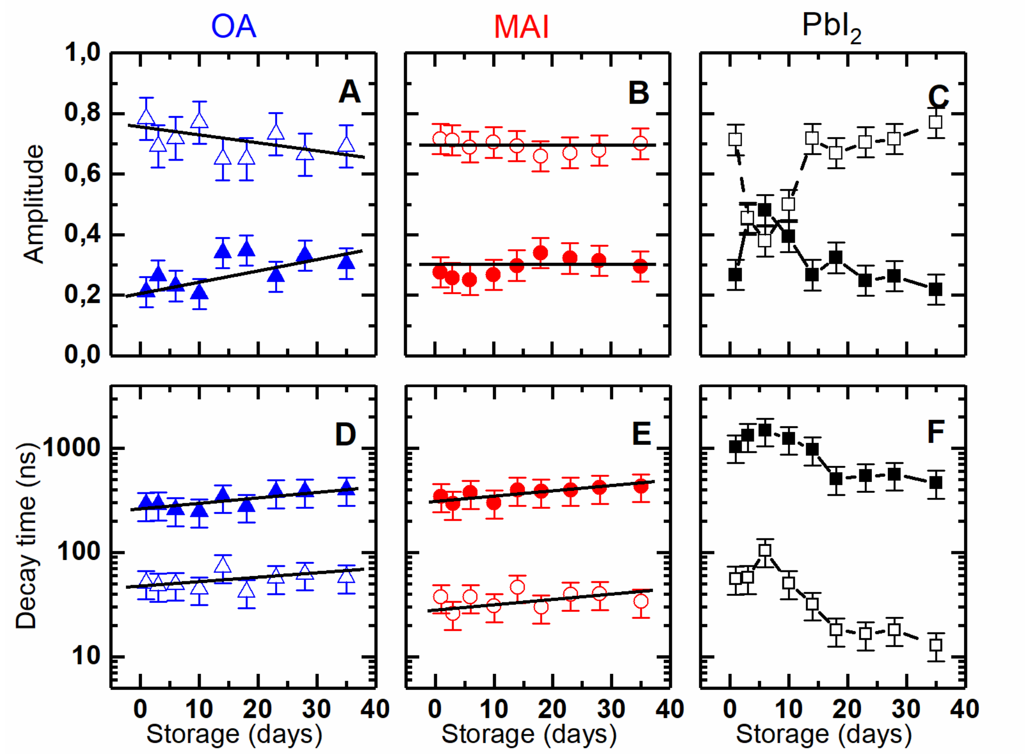

Figure S3. Colloidally exchanged QDs PL decay components evolution in porous matrix (black squares—PbI_2-treated QDs, red circles—MAI-treated QDs, blue triangles—OA-capped QDs). Open symbol stands for relaxation from the bright state while solid symbol stands for relaxation from the dark state. Black lines are given as a guide to the eye.

Table S1. Shifts of spectral PL parameters during 35-day storage in ambient conditions.

Shell Type	Solution PL Peak Shift, meV	Solid PL Peak Shift, meV	Solution PL FWHM Shift, meV	Solid PL FWHM Shift, meV
Oleic acid	20	45	10	35
PbI_2	65	90	15	120
MAI	25	60	5	40
